# Correlation of Blood FoxP3+ Regulatory T Cells and Disease Activity of Atopic Dermatitis

**DOI:** 10.1155/2019/1820182

**Published:** 2019-09-15

**Authors:** Yan Li, Wei Xu, Jingyi Yao, Haiyan Cheng, Xiaoli Sun, Linfeng Li

**Affiliations:** ^1^Department of Dermatology, Beijing Friendship Hospital, Capital Medical University, Beijing 100050, China; ^2^Research Center of Beijing Friendship Hospital, Capital Medical University, China; ^3^Beijing Institute of Clinical Medicine, China; ^4^Beijing Key Laboratory of Transplant Tolerance and Organ Protection, Beijing 100050, China

## Abstract

**Objectives:**

To investigate CD4+CD25+FoxP3+ T regulatory cells (Tregs) in the peripheral blood of patients with atopic dermatitis (AD) and its correlation with disease severity.

**Methods:**

Blood samples from 79 AD patients before and after four-week conventional treatment were collected. Cell counts of CD4+CD25+FoxP3+Tregs, CD4+CD25+FoxP3-T effector cells (Teffs), and CD4+IL-10+Tregs were analyzed by flow cytometry. Serum levels of IL-4, IL-10, IL-12, IL-13, IFN-*γ*, and TGF-*β* were measured by ELISA.

**Results:**

The pretreatment cell count of CD4+CD25+FoxP3+Tregs positively correlated with disease severity in all patients (*P* < 0.0001). However, when that correlation was rechecked based on the treatment response, a much stronger correlation of that was found in those patients with remission after treatment, while no correlation of that was found in patients without remission. Both the cell count and proportions of peripheral CD4+CD25+FoxP3+Tregs and CD4+CD25+FoxP3-Teffs reduced significantly after treatment in patients with remission, but remained unchanged in patients without remission. The cell count and proportion of CD4+IL-10+Tregs did not change after treatment in both groups. In patients with remission, serum levels of IL-4 and IL-13 significantly reduced (all *P* < 0.05); IL-12 and IFN-*γ* levels increased significantly (all *P* < 0.05); IL-10 and TGF-*β* levels remained unchanged after treatment. None of those cytokine levels changed in patients without remission.

**Conclusions:**

CD4+CD25+FoxP3+Tregs is associated with AD development and severity in some patients but not in others. AD maybe divided into CD4+CD25+FoxP3+Treg-associated subtype, which CD4+CD25+FoxP3+Treg is parallel to the activity of AD, and nonassociated subtype, which CD4+CD25+FoxP3+Treg is not related. This subgroup difference may contribute partly to the nonidentical markers that have been found in AD and should be studied further.

## 1. Introduction

Atopic dermatitis (AD), also called atopic eczema, is a chronic, recurrent, and inflammatory skin disease. It is characterized by severe pruritus, eczema-like lesions, and dry skin [1]. The etiology of AD is complex. In spite of intensive and extensive efforts in research, the exact etiology remains unclear. Recent studies suggest that it may be associated with immune disorder, such as a Th1/Th2-associated chemokine imbalance and skin barrier dysfunction [2, 3]. In addition, patient genetics and environment factors could also contribute to the initiation and development of AD. Most research on the etiology of AD emphasizes the correlation between immune disorders and AD. Previous studies have shown that immunoglobulin E- (IgE-) mediated allergic reaction results in inflammatory skin responses that are similar to the skin lesions of AD [4]. Disruption of the homeostasis of the effector T cells (Teffs) and regulatory T cells (Tregs) is considered a critical etiological factor for AD. It is well accepted that Tregs regulate autoimmune response and play essential roles in many autoimmune, allergic, and inflammatory diseases [5]. Tregs can prevent excessive immune response by inhibiting the activity and proliferation of Teffs so to enhance immune tolerance and maintain immune homeostasis. Thus, Tregs are critical for maintaining peripheral tolerance, preventing autoimmune diseases, and limiting inflammatory responses [5, 6]. Tregs include natural Tregs and induced Tregs. CD4+CD25+FoxP3+Tregs belong to natural Tregs and overexpress forkhead box P3 (Foxp3), which is essential for CD4+CD25+Treg function. Activated CD4+CD25+Tregs can release anti-inflammatory cytokines, such as TGF-*β* and IL-10. Although the cytokines may be involved in the function of CD4+CD25+Tregs, they are not required for CD4+CD25+Tregs-mediated regulation of immune function; instead, a direct cell-cell contact is required [6, 7]. CD4+CD25+FoxP3+Tregs inhibit the function of antigen-presenting cells (APC), effector T cells, and mast cells [5]. Induced Treg such as Tr1 cells overexpress IL-10. Although these T cells do not express Foxp3, they can upregulate Foxp3 after being activated. CD4+IL-10+Tregs release IL-10 to inhibit the proliferation of Th1 and Th2 cells [8]. Natural Tregs and induced Tregs have very different gene expression profiles and different functions [9]. The specific types, quantity, and precise function of Tregs in patients with AD are still unclear [10], and previous findings on Tregs of patients with AD are controversial [11–13]. To investigate the association between AD and CD4+CD25+FoxP3+Tregs, we collected peripheral blood samples from patients with moderate to severe AD before and after treatment and analyzed Treg characteristics.

## 2. Materials and Methods

### 2.1. Study Design

This was a prospective observational cohort study. The study protocol has been approved by the Institutional Review Board of Beijing Friendship Hospital (Approval No. 2015-P2-066-01). Written informed consent was obtained from each study participant.

### 2.2. Patients

Consecutive patients with moderate to severe AD treated in the Department of Dermatology of Beijing Friendship Hospital from January 1 to December 31, 2016 were included. The diagnosis of AD was confirmed using the UK Working Party's diagnostic criteria. The severity of AD was assessed based on the Rajka and Langeland criteria. At least 10% body surface was affected by the disease. Patients with the eczema area and severity index (EASI) [14] between 7 and 14 were considered moderate to severe AD. Patients, who were pregnant, had autoimmune diseases, chronic systemic disease, and/or other skin diseases, or had a history of tumor or familial tumor diseases were excluded.

All patients were treated with conventional therapies for AD, including antihistamines such as cetirizine tablets, loratadine, and mizolastine, compound glycyrrhizin capsules, topical mometasone furoate cream, and emollients for four weeks. Treatment effectiveness index = (pretreatment EASI − posttreatment EASI)/pretreatment EASI × 100%. Complete cure was defined as treatment effectiveness index ≥ 90%. Significant improvement was defined as 60% ≤ treatment effectiveness index < 90%. Moderate improvement was defined as 20% ≤ treatment effectiveness index < 60%. No improvement was defined as treatment effectiveness index < 20% [15]. Patients with complete cure or significant improvement were considered to have AD remission. Patients with moderate improvement or no improvement were considered to have active AD.

### 2.3. Flow Cytometry

Peripheral venous blood was collected from patients before and after treatment. Peripheral blood mononuclear cells (PBMCs) were extracted from the blood samples and stored at -80°C. After all samples collected, PBMCs were washed twice with DPBS (Corning, USA) and then resuspended in RPMI 1640 (Corning, USA) with 2% FBS (Gibco, USA). Then, the suspended cells were stimulated by cell stimulation cocktail (eBioscience, USA) for 4 hours in 37°C with 5% CO_2_. After stimulation, cells were washed and incubated with anti-CD4-FITC, anti-CD4-Percp, anti-CD25-PE, and anti-CD127-FITC. All the aforementioned reagents were purchased from BD Biosciences (USA). Intracellular IL-10-PE staining was performed using Transcription Factor Buffer Set (BD Biosciences, USA), and FOXP3-647 staining was performed using the Human FoxP3 Buffer Set (BD Biosciences, USA) according to the manufacturer's instructions. Data were collected using a four-color FACSCalibur™ flow cytometer (BD Biosciences, USA) and analyzed using FlowJo software version 10.1 (Tree Star, Ashland, OR, USA).

### 2.4. ELISA

The levels of IFN-*γ*, IL-4, IL-10, IL-12, IL-13, and TGF-*β* in the serum samples were analyzed by ELISA using ELISA kits. The ELISA kits for human IL-4 (Catalog #KHC0041), human IL-10 (Catalog #KHC0101), human IL-12 p40/p70 (Catalog #KHC0121), human IL-13 (Catalog #KHC0131), and Human IFN-*γ* (Catalog #KHC4021) were from Invitrogen (USA). The ELISA kit for human TGFB2 was from Thermo Scientific (USA). ELISA reader (Spectra Max M3) was from Molecular Devices (USA).

### 2.5. Statistical Analysis

Descriptive statistics were used to detail the baseline characteristics. Mean with standard deviation (SD) and range was used for continuous variables (age, disease history, and EASI before and after treatment). Absolute number was used for categorical variables (gender). A paired *t*-test was used to compare different types of T cells (CD4+CD25+FoxP3+Tregs, CD4+CD25+FoxP3-Teffs, CD4+IL-10+Tregs) and cytokine levels before and after treatment of the AD remission and active groups. The association between CD4+CD25+FoxP3+Tregs count and pretreatment EASI for the two groups was analyzed by Pearson's correlation test.

All statistical analyses were conducted using SPSS version 16.0. *P* < 0.05 was considered statistically significant.

## 3. Results

### 3.1. General Clinical Data

A total of 79 patients with moderate to severe AD were included. Of the 79 patients (aged 19-40 years, 33 men), 40 became AD remission after treatment; 39 remained active AD after treatment. Patients' general clinical data are presented in Table 1. The mean age of the 79 patients was 25.06 ± 5.20 years. Demographics and pretreatment EASI were similar in the remission and active groups (Table 1).

### 3.2. Comparison of Different Types of T Cell Counts before versus after Treatment

**Figure 1:**
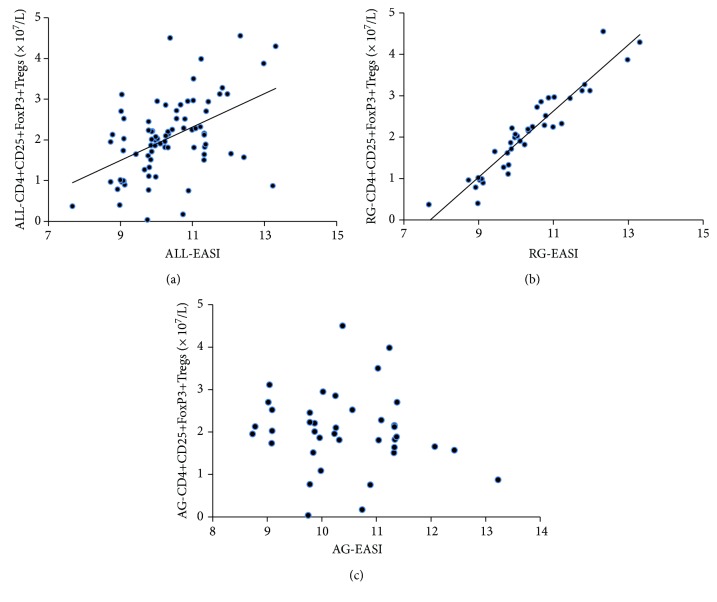
Correlation between pretreatment CD4+CD25+FoxP3+Treg count and EASI. (a) The pretreatment cell count of CD4+CD25+FoxP3+Tregs positively correlated with their pretreatment EASI in all AD patients. (b) Patients in the AD remission group (RG) showed significant linear correlation between their pretreatment CD4+CD25+FoxP3+Treg count and pretreatment EASI. (c) No such linear correlation in the AD active group (AG).

**Figure 2:**
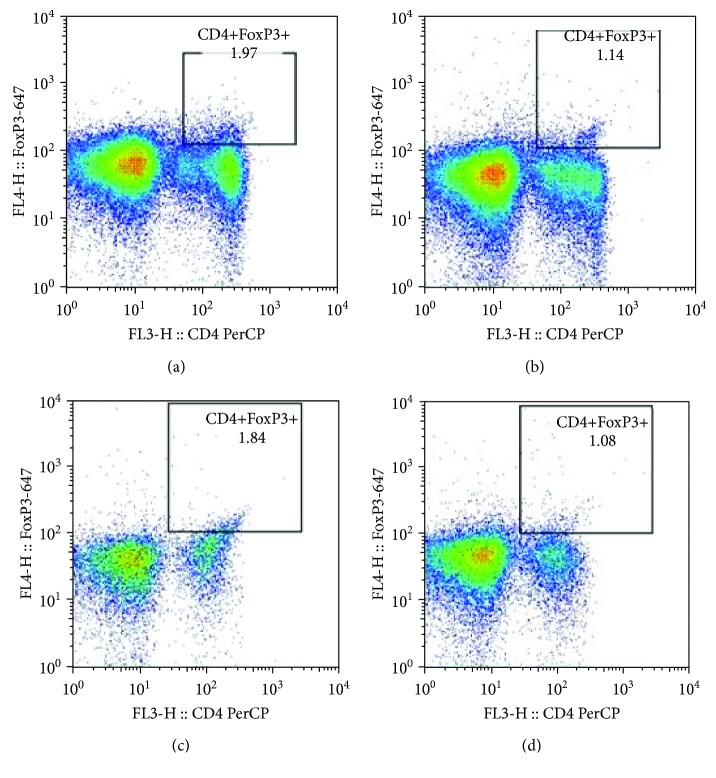
Representative scatter plot of CD4+CD25+FoxP3+Treg analysis. The pretreatment (a) and posttreatment (b) scatter plot of a representative patient from the AD remission group. The pretreatment (c) and posttreatment (d) scatter plot of a representative patient from the AD active group.

**Figure 3:**
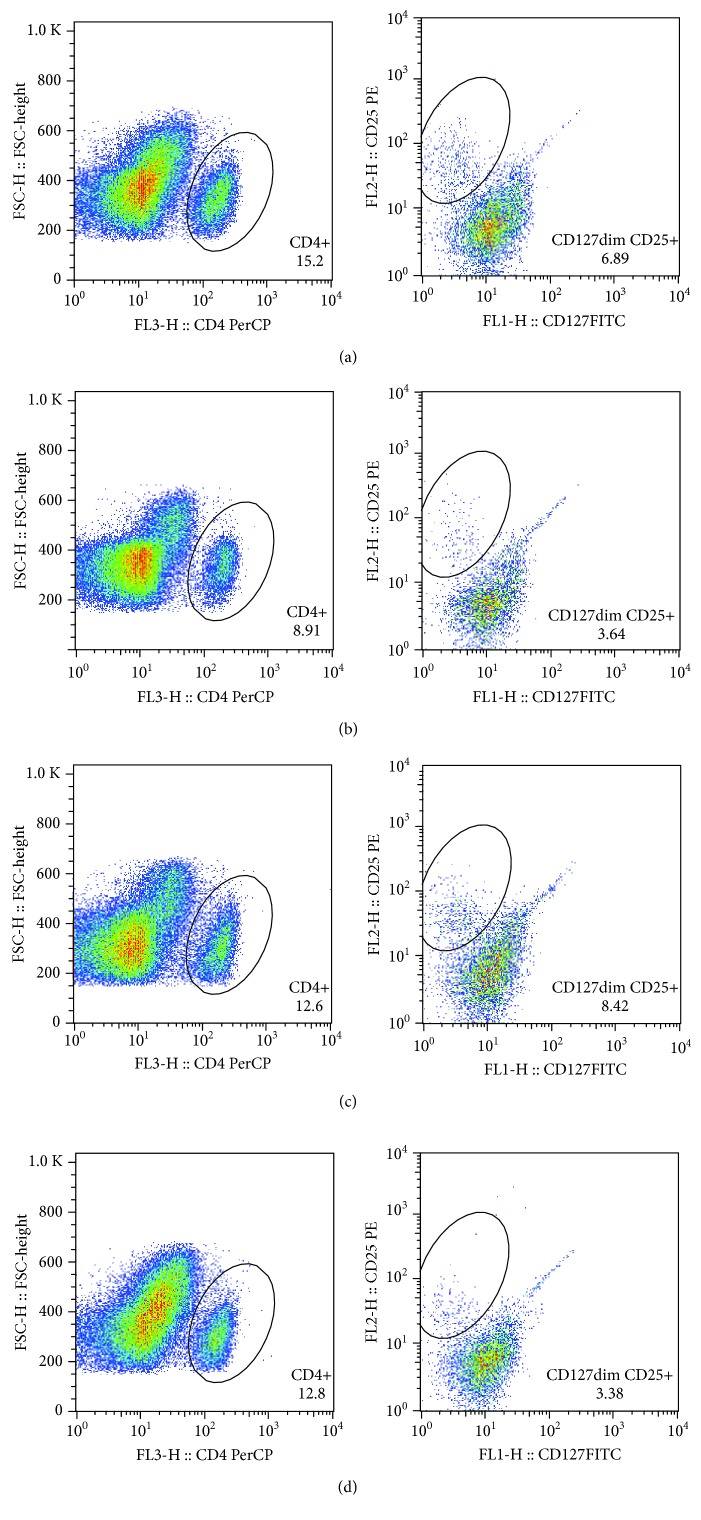
Representative scatter plot of CD4+CD25+FoxP3-Teff analysis. The pretreatment (a) and posttreatment (b) scatter plot of a representative patient from the AD remission group. The pretreatment (c) and posttreatment (d) scatter plot of a representative patient from the AD active group.

**Figure 4:**
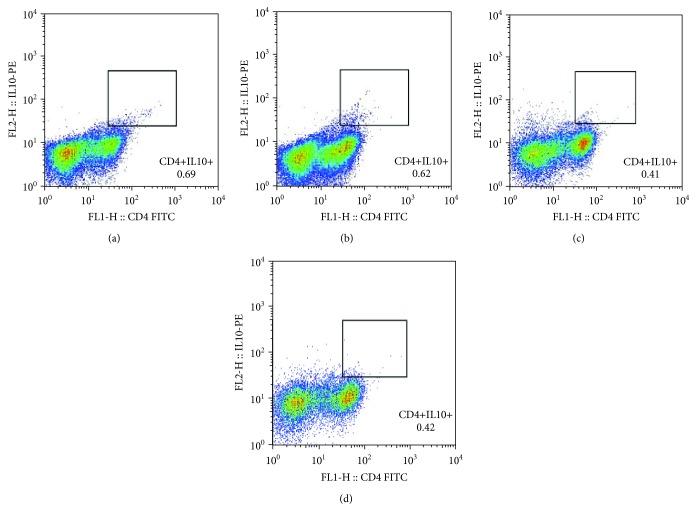
Representative scatter plot of CD4+IL-10+Treg analysis. The pretreatment (a) and posttreatment (b) scatter plot of a representative patient from the AD remission group. The pretreatment (c) and posttreatment (d) scatter plot of a representative patient from the AD active group.

The pretreatment cell count of CD4+CD25+FoxP3+Tregs positively correlated with their pretreatment EASI in all AD patients (*r* = −0.482, *P* ≤ 0.001, Figure 1(a)). Subsequently, these AD patients were divided into remission group and still active group according to their response to the treatment effect. Notably, the pretreatment CD4+CD25+FoxP3+Treg cell count of patients from the AD remission group linearly correlated to their pretreatment EASI significantly (*r* = 0.95, *P* < 0.0001, Figure 1(b)), whereas no such correlation was observed in patients from the AD active group (Figure 1(c)). Mean cell count and proportions of different types of T cells are presented in Table 2. For patients in the AD remission group, both the absolute cell counts and the proportions of CD4+CD25+FoxP3+Tregs and CD4+CD25+FoxP3-Teffs reduced significantly after treatment (all *P* < 0.05, Table 2). In contrast to the AD remission group, patients in the AD active group showed similar cell counts and proportions of those T cells before and after treatment (Table 2). Moreover, the cell count and proportion of CD4+IL-10 Tregs were not significantly changed by treatment in both AD remission and active groups (Table 2). Representative scatter plots of analyses for CD4+CD25+FoxP3+Tregs (Figure 2), CD4+CD25+FoxP3-Teffs (Figure 3), and CD4+IL-10+Tregs (Figure 4) are displayed in Figures 2–4.

### 3.3. Comparison of Cytokine Levels in Serum

**Figure 5:**
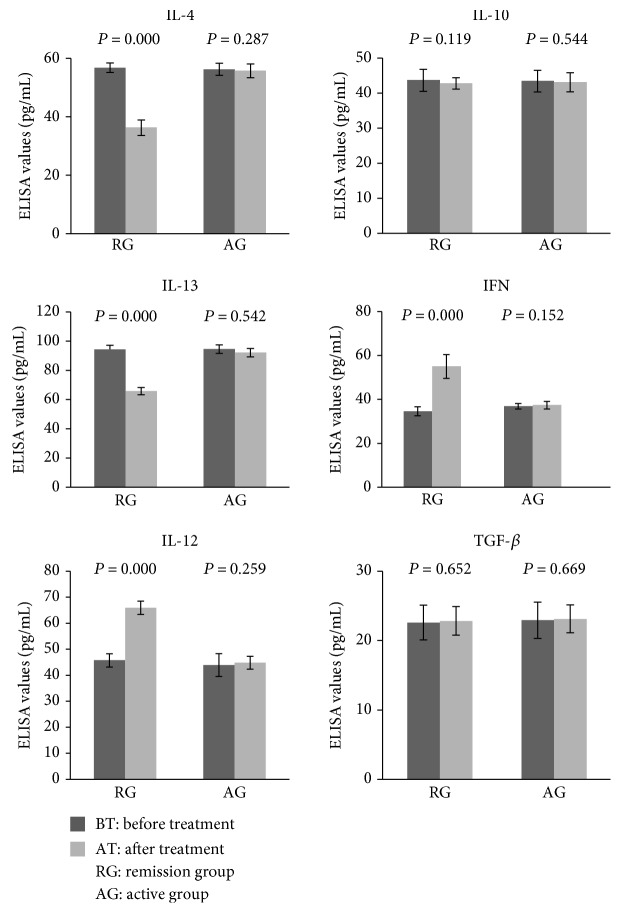
Comparison of cytokine levels in serum before versus after treatment. Cytokines in serum samples were analyzed by ELISA. A paired *t*-test was used to compare cytokine levels before versus after treatment.

For patients in the AD remission group, compared with the values before treatment, posttreatment serum levels of IL-4 and IL-13 reduced significantly (all *P* < 0.05), IFN and IL-12 levels increased significantly (all *P* < 0.05), and IL-10 and TGF-*β* levels remained unchanged (Figure 5). In contrast to the AD remission group, none of those cytokines were significantly changed after treatment in patients from the AD active group (Figure 5).

## 4. Discussion

The current study found that the pretreatment number of CD4+CD25+FoxP3+Tregs was associated with AD severity and reduced significantly after treatment in the remission group, whereas the active group did not show this association. These findings suggest that AD may have a CD4+CD25+FoxP3+Treg-associated subtype and a nonassociated subtype.

Previous studies on CD4+CD25+Tregs in AD have shown controversial results. In 2004, Ou et al. reported that the cell count of CD4+CD25+Tregs in the peripheral blood of patients with AD was twice of that of the normal control group [11]. However, in 2009, the two independent research groups Szegedi et al. and Brandt et al. found that the cell count of CD4+CD25+FoxP3+Tregs of patients with AD was not different from that of the healthy controls [16, 17]. Notably, more recent studies demonstrated that patients with AD showed increased CD4+CD25+FoxP3+Treg cell count and the cell count positively correlated with AD severity [10, 11, 18, 19]. Our results showing the posttreatment reduction in the numbers of CD4+CD25+FoxP3+Tregs are consistent with those of the more recent studies. We believe the conflicting findings may indeed reflect the complex roles of CD4+CD25+FoxP3+Tregs in the initiation and development of AD. Our results also indicate that CD4+CD25+FoxP3+Tregs could be involved in AD development only in some patients. Elevation of circulating Tregs could be a compensatory mechanism for the severe inflammation of patients with AD [20].

Tregs induce, maintain, and inhibit Th1/Th2 immune responses via Th1-type and Th2-type cytokines [21]. Thus, we determined these cytokine levels in serum. IL-4 and IL-13 can stimulate B cell proliferation and IgE synthesis so to induce T cells to differentiate into Th2 cells and inhibit Th1 differentiation. We found that serum IL-4 and IL-13 levels were reduced after treatment in the remission group. Thus, IL-4 and IL-13 may be the essential Th2-type cytokines to induce AD and play critical roles in the etiology of acute and chronic AD in some patients [1, 22, 23]. IFN-*γ* and IL-12, which are mainly secreted by Th1 cells and APCs, can induce NK and T cell proliferation, promote T and NK cell-mediated cytotoxicity, stimulate Th0-to-Th1 differentiation, regulate Th1 cell proliferation, and inhibit IgE synthesis and Th2 function. Our current study showed serum IFN-*γ* and IL-12 levels were increased after treatment in the remission group. Previous studies on serum IL-10 levels in patients with AD show inconsistent results. Vakirlis et al. found that serum IL-10 levels in patients with AD were lower than those in the healthy controls [24]. However, Lesiak et al. reported that PBMCs of patients with AD produced more IL-10 than the PBMCs of healthy controls [25]. Hussein et al. found no significant difference in serum IL-10 levels between patients with AD and the healthy controls [26]. We found no changes in serum IL-10 after treatment in our patients. The inconsistent results on serum IL-10 levels in AD may indirectly reflect the complex mechanism underlying IL-10 production in the human body. In addition to Th2 cells, Th0, Trl, and B cells also can release IL-10 [27].

The proportion of peripheral CD4+CD25+Tregs in total CD4+T cells in the blood of a healthy person is 5%-10% [28]. Approximately 97% of Foxp3 is expressed in CD4+CD25+Tregs. Our current study showed that in patients with AD remission, the proportion of CD4+CD25+FoxP3+Tregs before and after treatment was 16.97% and 10.72%, respectively, indicating the abnormal immune function in patients with AD. In addition, both the pretreatment and posttreatment CD4+CD25+FoxP3+Treg percentages of this current study were higher than the previously reported normal range [28]. These findings appear to be opposite to some previous reports showing that CD4+CD25+FoxP3+Treg proportions were lower in patients with AD than in healthy persons [29, 30]. During the development of AD, elevation of circulating CD4+CD25+FoxP3+Tregs could possibly inhibit severe inflammation in patients with AD. Tregs can inhibit Teff activation via a direct cell-cell contact and cytokines and can also downregulate immune response via the anti-inflammatory cytokines IL-10 and TGF-*β*. The current study showed that in patients with posttreatment remission, their cell counts of both CD4+CD25+FoxP3+Tregs and CD4+CD25+FoxP3-Teffs were higher at disease active stage than at remission stage. These results indicate that immune reactions may reduce Treg-mediated inhibition on Teffs as AD progresses, resulting in Teff elevation [28]. Both pretreatment and posttreatment CD4+IL-10+Treg proportions of this current study were higher than the normal range (0.01 × 10^9^‐0.03 × 10^9^/L), suggesting that CD4+IL-10+Tregs could be involved in the development of AD. Notably, we found that treatment did not affect CD4+IL-10+Treg cell counts in both remission and active groups, indicating that CD4+IL-10+Treg levels may not correlate with AD status.

The current study showed that in patients with remission, CD4+CD25+FoxP3+Tregs levels reduced after treatment but serum levels of IL-10 and TGF-*β*, which were mainly secreted by CD4+CD25+FoxP3+Tregs, were not changed after treatment. These results may indicate that the function of CD4+CD25+FoxP3+Tregs could be changed during the development of AD. At AD onsets, CD4+CD25+FoxP3+Tregs may be activated and transported to the target tissue and differentiated. However, their function could be inhibited as AD exacerbates [5]. In patients with active AD after treatment, all the three types of Tregs were not affected by treatment, further implying the heterogeneity of AD.

The pretreatment cell count of CD4+CD25+FoxP3+Tregs positively correlated with their pretreatment EASI in all AD patients (*r* = −0.482, *P* ≤ 0.001). Similarly, previous studies have also found a positive linear correlation between the cell count of CD4+CD25+FoxP3+Tregs and disease severity [10, 11, 18, 19]. Subsequently, these AD patients were divided into remission group and active group according to the treatment effect. In the current study, we found that in patients with posttreatment remission, pretreatment cell count of CD4+CD25+FoxP3+Tregs and EASI had a positive linear correlation, indicating that severer skin lesions appear to be associated with more CD4+CD25+FoxP3+Tregs. However, we found no such correlation in patients with active AD after treatment. These findings further reflect the complexity of the etiology of AD. CD4+CD25+FoxP3+Tregs may be involved in AD development in some patients but not in others. We take the view this explains why the results of quite a number of articles appear controversial, or even completely opposite. The reason may very likely be that the target patients have different pathogenesis. AD may be classified into multiple subtypes when diagnosis is based on clinical symptoms. No common biomarkers of AD have been found yet. Patients diagnosed with AD can have very different pathogenic mechanisms. Currently, AD is classified into exogenous AD and endogenous AD. Our current study also supports the diversity of AD. After all, AD with different pathogenesis could be different diseases and should be treated differently.

In summary, our current study demonstrated that the pretreatment number of CD4+CD25+FoxP3+Tregs was positively associated with AD severity and reduced significantly at AD remission in patients with posttreatment remission but not in patients with posttreatment active AD. These data suggest that AD may be classified into a CD4+CD25+FoxP3+Treg-associated subtype and a nonassociated subtype.

Patients with CD4+CD25+FoxP3+Treg-associated subtype may benefit from conventional treatments better than those with nonassociated subtype.

If this inference could be set up, it will be possible to estimate the curative effect of the AD patients who have been treated with traditional therapy for a period of time by calculating the correlation EASI scores and the number of CD4+CD25+FoxP3+Tregs before and after treatment for these patients. It will be possible to monitor the conditions of CD4+CD25+FoxP3+Treg-associated subtype patients by regularly testing the number of CD4+CD25+FoxP3+Tregs. The main limitation of this study is the relatively small sample size. Large-scale studies are required to further verify the conclusions.

## Figures and Tables

**Table 1:** General clinical data.

	AD remission after treatment (*N* = 40)	AD active after treatment (*N* = 39)
Men/women	13/27	20/19
Age		
Range, min–max (years)	20-38	19-40
Mean ± SD (years)	25.25 ± 5.19	24.87 ± 5.27
Disease history (months)		
Range, min–max (months)	2-85	3-76
Mean ± SD (months)	19.18 ± 20.56	18.92 ± 15.33
EASI		
Before treatment		
Range, min–max	7.67-13.31	8.73-13.23
Mean ± SD	10.29 ± 1.19	10.43 ± 1.04
After treatment		
Range	2.23-4.98	5.98-8.45
Mean ± SD	3.61 ± 0.64	6.80 ± 0.60

**Table 2:** Comparison of different types of T cell counts before versus after treatment.

	AD remission after treatment (*N* = 40)			AD still active after treatment (*N* = 39)		
	Before	After	*P*	Before	After	*P*
CD4+CD25+FoxP3+Tregs						
Absolute count (×10^7^/L)	2.06 ± 1.00	1.03 ± 0.45	<0.001	2.04 ± 0.90	2.05 ± 0.96	0.965
% in CD4+ cells (%)	16.97 ± 5.01	10.72 ± 4.87	<0.001	16.76 ± 4.55	16.77 ± 4.07	0.994
CD4+CD25+FoxP3-Teffs						
Absolute count (×10^7^/L)	9.78 ± 3.35	8.10 ± 3.80	0.034	9.98 ± 3.92	9.97 ± 4.72	0.993
% in CD4+ cells (%)	82.65 ± 11.55	76.58 ± 12.06	0.02	82.03 ± 17.79	81.32 ± 19.11	0.813
CD4+IL-10+Tregs						
Absolute count (×10^9^/L)	0.26 ± 0.20	0.22 ± 0.29	0.501	0.26 ± 0.34	0.26 ± 0.30	0.941
% in CD4+ cells (%)	12.44 ± 0.42	12.36 ± 0.38	0.336	12.45 ± 0.54	12.42 ± 0.38	0.804

## Data Availability

The data used to support the findings of this study are included within the article.
